# Epigallocatechin‐3‐Gallate Mitigates Atopic Dermatitis‐Like Skin Lesions and Psychiatric Comorbidities by Reducing Oxidative Stress

**DOI:** 10.1002/fsn3.71248

**Published:** 2025-11-23

**Authors:** Liu Tang, Ruzhen Peng, Jingyi Guo, Chun Wang, Tianshuai Wang, Yindi Bao, Ying He

**Affiliations:** ^1^ Department of Pharmacy Renmin Hospital of Wuhan University Wuhan China; ^2^ The Key Laboratory of Anti‐Inflammatory and Immune Medicine, Ministry of Education Anhui Medical University Hefei China; ^3^ Anhui Provincial Key Laboratory of Chinese Medicinal Formula Anhui University of Chinese Medicine Hefei China; ^4^ Hubei Key Laboratory of Wudang Local Chinese Medicine Research Hubei University of Medicine Shiyan China; ^5^ Department of Obstetrics and Gynecology Renmin Hospital of Wuhan University Wuhan China; ^6^ Department of Stomatology Renmin Hospital of Wuhan University Wuhan China

**Keywords:** atopic dermatitis, epigallocatechin gallate, Keap1/Nrf2/HO‐1 signaling pathway, oxidative stress, psychiatric comorbidities

## Abstract

Atopic dermatitis (AD), a chronic, relapsing, inflammatory skin disease often accompanied by psychiatric comorbidities like anxiety and depression, poses significant therapeutic challenges. This study aims to explore the therapeutic effects and mechanisms of dietary supplementation with epigallocatechin‐3‐gallate (EGCG) on AD skin lesions and comorbid anxiety/depressive disorders through in vivo and in vitro experiments. Macroscopical and histopathological evaluation revealed that gavage administration of EGCG (50 or 100 mg/kg) significantly improved AD skin lesions, as evidenced by the reduced dermatitis severity scores, TEWL value and blood flow perfusion, and diminished infiltration of inflammatory and mast cells (*p* < 0.05 or *p* < 0.01). In EPM and OFT, EGCG‐treated mice displayed markedly decreased anxiety and depressive‐like behaviors, as manifested by the increased frequency of entries and distance traveled in the open arms and the central area, and elevated 5‐HT levels in serum/brain (*p* < 0.05 or *p* < 0.01). Additionally, compared to the AD model group, EGCG administration reduced MDA content and ROS accumulation in serum/brain/skin, while enhancing GSH‐Px and CAT activity (*p* < 0.05 or *p* < 0.01). Flow cytometry analysis further demonstrated that EGCG could suppress the generation of ROS in TNF‐α/IFN‐γ‐stimulated NHEK cells. Mechanistically, by virtue of in vivo, in vitro and in silico assays, the results of western blot, immunofluorescence and molecular docking discovered that EGCG could promote Nrf2 nuclear translocation and ultimately reduce oxidative stress, at least in part, by specifically targeting Keap1 to disrupt the interaction of Keap1/Nrf2 and modulation of the Keap1/Nrf2/HO‐1 axis. These aforementioned findings highlight the potential of EGCG as a promising therapeutic strategy for AD and its psychiatric comorbidities by alleviating Keap1/Nrf2/HO‐1‐mediated oxidative stress.

## Graphical abstract

Administration of EGCG attenuates atopic dermatitis (AD)‐like skin manifestations. EGCG ameliorates AD‐associated behavioral deficits by altering the HPA axis dysfunction and upregulating the neurotransmitters. Down‐regulation of Keap1/Nrf2/HO‐1 signaling pathway‐mediated oxidative stress might be the crucial mechanism for EGCG against AD‐like phenotype accompanied by psychological disorders.

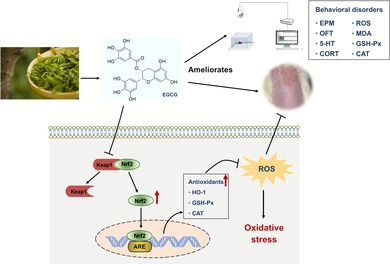

## 1 Introduction

Atopic Dermatitis (AD), a chronic, relapsing inflammatory skin disorder, has a significant global prevalence among children and adults. The hallmark symptoms of AD encompass dry skin, intense pruritus, and eczematous skin lesions, which not only impact patients' physical health but also severely affect their mental well‐being and quality of life (Kim et al. 2019; Schuler 4th et al. 2023). The pathogenesis of AD is intricate, with interplay between genetic, environmental, immunological, and psychological elements. Studies have shown that psychological stress states, such as anxiety and depression, exacerbate pruritus in AD patients. Conversely, AD symptoms adversely affect patients' psychological states, engendering a vicious cycle (Gonzalez‐Uribe et al. 2023; Paller et al. 2018). This interrelationship underscores the imperative for therapeutic approaches that can concurrently address both skin manifestations and psychological conditions.

Reactive oxygen species (ROS) are naturally generated during routine metabolic activities within the body. When the body's antioxidant defenses are overwhelmed and unable to counterbalance ROS production, an excessive buildup of ROS occurs in cells, leading to oxidative stress. Indeed, a recent finding highlighted that ROS derived from keratinocytes can activate type 2 inflammation and intense pruritus in AD skin (Choi et al. 2021). Clinicopathological data from AD patients reveal elevated levels of ROS and oxidative stress markers, uncovering the pivotal role of oxidative stress in the pathogenesis of AD (Khan et al. 2022). Supplementation of exogenous antioxidants to redress the imbalance of oxidative stress and counteract the excess ROS in AD has emerged as a widely accepted and effective approach for AD.

Epigallocatechin‐3‐gallate (EGCG), the predominant catechin found in green tea (Camellia sinensis L.), accounts for over 50% of the total green tea polyphenols. Researchers have documented that EGCG is responsible for the majority of the potential health benefits attributed to green tea consumption, including cancer chemoprevention, anti‐inflammatory, antioxidant, and skin barrier‐protective effects (Messire et al. 2023; Singh et al. 2011), shedding light on EGCG's prospective role in AD therapy. Moreover, EGCG has demonstrated considerable antidepressant‐like effects by augmenting hippocampal serotonin (5‐HT) levels and inhibiting the overactivation of the hypothalamic–pituitary–adrenal (HPA) axis and neuroinflammation (Abdelmeguid et al. 2022; Li et al. 2020). Accordingly, we hypothesize that EGCG might mitigate the AD‐like phenotype accompanied by psychological disorders by modulating oxidative‐stress signaling cascades and ROS generation.

To address this question, the therapeutic effects of EGCG against AD were evaluated using a 2,4‐dinitrofluorobenzene (DNFB)‐induced mouse model, with particular focus on animal behaviors, biochemical parameters, skin pathology, SCOARD, and skin barrier function. Additionally, an in silico analysis with an in vitro AD model of TNF‐α/IFN‐γ‐stimulated NHEK cells was performed to further unravel the cellular mechanisms.

## 2 Materials and Methods

### 2.1 Materials

EGCG was bought from Shanghai Aladdin Bio‐Chem Technology Co. Ltd., China. DNFB (purity > 99%) was supplied by Shanghai Adamas Reagent Co. Ltd., China. All other general agents were commercially available.

### 2.2 Animals and Grouping

A total of 32 female Kunming mice, aged 8 weeks and weighing between 19 and 21 g, were supplied by the Laboratory Animal Center of Wuhan University. These mice were accommodated in the university's Animal Care Facility, adhering to the institution's protocols for the care and use of laboratory animals. The study's plans involving the animals received clearance from the Ethics Committee of Wuhan University, China, with the Permit Number 42010200006107, and all experimental procedures were conducted in accordance with the regulations set by the State Council of the People's Republic of China for the management of experimental animal affairs.

For the establishment of the AD model and subsequent treatments, mice were grouped using a random number table method, and evenly divided into four distinct groups, each consisting of eight animals: (1) the Normal group; (2) the DNFB‐induced AD model group; (3) DNFB+EGCG low dose (50 mg/kg/day) group; (4) DNFB+ EGCG high dose (100 mg/kg/day) group. The induction of AD‐like symptoms in the mice was achieved through repeated applications of DNFB to the dorsal skin, which has been reported in our previous studies (Gao et al. 2022; Tang et al. 2022). EGCG was dissolved in sterile 0.9% saline to a concentration of 50 or 100 mg/kg and administered orally to the DNFB+EGCG groups at a dosage volume of 0.1 mL/10 g body weight. The normal and AD model groups received equal volumes of saline. The experimental procedure is detailed in Figure S1.

### 2.3 Evaluation of Dermatitis Severity Score, Skin Swelling and TEWL

The assessment of dermatitis severity was conducted macroscopically, taking into account the four primary clinical features of AD as follows (Yun et al. 2010): 0 for no symptoms, 1 for mild, 2 for moderate, and 3 for severe symptoms across four categories: erythema/hemorrhage, scaling/dryness, edema/swelling, and erosion/excoriation.

Furthermore, after the mice were anesthetized, a MoorFLPI‐2 Laser Doppler Imager was employed to examine the blood perfusion in the depilated modeling area of dorsal skin (2 × 3 cm), which reflects the degree of erythema. Skin edema was assessed by measuring the variation in skin thickness or weight among the different groups. Upon completion of the experiments on Day 14, the dorsal skin from each group was excised and cut into circular sections with an 8 mm diameter punch. An analytical balance was utilized to determine the weight of the skin samples, while a vernier caliper was employed to measure skin thickness.

The measurement of TEWL (g/m^2^/h), an epidermal biomarker of the skin barrier, was quantified on Day 12 for each mouse in each group using the VAPO SCAN AS‐VT100RS device (manufactured in Asch, Japan). A cylindrical probe with a diameter of 12 mm was placed at the center of the shaved area on the mouse's dorsal skin for approximately 30 s. During the measurements, the ambient temperature was maintained at 25°C ± 5°C and relative humidity at 55% ± 5%.

### 2.4 Histopathological Examination

Dorsal skin tissue specimens were fixed in 4% formaldehyde, embedded in paraffin, and sectioned into 4‐μm thick slices for hematoxylin–eosin (H&E) staining and Toluidine blue (TB) staining. ImageJ software was used to measure the thickness of the epidermis and to quantify the number of mast cells.

### 2.5 Behavioral Tests

To explore the effect of EGCG on mental psychiatric disorders in AD mice, open field tests (OFT) and the elevated plus maze (EPM) were conducted, following procedures outlined in previous research (Dang et al. 2022; Wang et al. 2021). Behavioral measures were recorded for each mouse, and the apparatus was disinfected with 75% ethanol after each session.

#### 2.5.1 Open‐Field Test (OFT)

On Day 12, mice were introduced to an open‐field apparatus. The open field test was performed for 6 min with a square apparatus (50 cm × 50 cm × 50 cm) in which the area was divided into 25 equal squares. Following a 2‐min acclimation phase, the subjects' travel distance, velocity, periods of inactivity, and movement paths over a subsequent 4‐min trial were monitored and quantified utilizing the SuperMaze tracking apparatus (Shanghai, China).

#### 2.5.2 Elevated Plus Maze (EPM)

On Day 12 of the experiment, the EPM test was performed with a polypropylene plastic cruciform apparatus consisting of two open arms (15 cm × 40 cm), and two closed arms (15 cm × 40 cm), arranged in such a way that the two arms of each type are opposite to each other. All the arms are elevated 30 cm above the floor, and are surrounded with drapes. For the EPM test, one mouse was placed in the center of the maze facing one of the two closed arms for a 5‐min test. The number of entries and time spent in the open arms were measured with video tracking software.

### 2.6 Enzyme‐Linked Immunosorbent Assay (ELISA)

Blood samples were gathered and subjected to centrifugation at 1370 *g* for 15 min at 4°C to separate the serum. Skin tissues were weighed, processed into a homogenate, and then centrifuged at 15, 800 *g* for 15 min at 4°C to extract the supernatant for subsequent biochemical assays. The total serum level of immunoglobulin E (IgE), serum/skin/brain levels of oxidative stress markers, including catalase (CAT), glutathione peroxidase (GSH‐Px), and malondialdehyde (MDA), as well as serum/brain levels of neurotransmitters, such as 5‐HT and corticosterone (CORT), was quantified using ELISA kits, following the protocols provided by Quanzhou Ruixin Biotechnology Co. Ltd. The detection of ROS in serum/skin/brain levels of ROS was quantified by the kit (Cat. No. E004‐1‐1, Nanjing Jiancheng Bioengineering Institute) via a colorimetric reaction with 2,7‐dichlorofluorescin diacetate (DCFH‐DA), where units (U/mg protein) represent the relative ROS level normalized to protein concentration.

### 2.7 Cell Culture, Viability Assay and Sample Treatment

The normal human epidermal keratinocyte (NHEK) cells (Cat. No. HTX2253, Otwo biotech, Shenzhen, China) were cultured in Dulbecco's Modified Eagle Medium (DMEM) with high glucose levels, enriched with 10% fetal bovine serum (FBS) and antibiotic protection consisting of 100 U/mL penicillin and 100 μg/mL streptomycin. The cells were maintained at 37°C within a humidified atmosphere containing 5% CO_2_. For assessing cell viability, NHEK cells treated with/without TNF‐α/IFN‐γ (each 10 ng/mL) were plated at a concentration of 1 × 10^4^ cells per well in a 96‐well plate and exposed to various concentrations of EGCG ranging from 1 to 200 μM for a period of 24 h. The Cell Counting Kit‐8 (CCK8) assay was employed to determine the viability of EGCG‐exposed cells, following the manufacturer's protocol.

Furthermore, NHEK cells were seeded at a density of 5 × 10^5^ cells/well in 6‐well plates. The following day, cells were pre‐incubated with/without EGCG (5, 10, 25 μM) for 12 h, then treated with or without TNF‐α /IFN‐γ for 24 h. Subsequently, the cells were conducted for the detection of intracellular ROS levels and Keap1/Nrf2/HO‐1 signaling pathway‐associated proteins.

### 2.8 Detection of Cellular ROS Content by Flow Cytometry

ROS detection was performed using the fluorescent probe DCFH‐DA from the ROS Assay Kit (Beyotime, China), strictly following the manufacturer's protocols. After establishing the negative vehicle group and EGCG‐treated groups (5, 10, 25 μM), the cells except for the negative vehicle group were incubated with 10 μM 2′,7′‐dichlorodihydrofluorescein diacetate (DCFH‐DA) for 20 min at 37°C, followed by three washes with PBS to remove unbound probe. Subsequently, cells were harvested via trypsinization and centrifugation, and fluorescence intensity was analyzed using a flow cytometer (BD FACScelesta, USA) at excitation/emission wavelengths of 488/525 nm, with data processed by FlowJo software.

### 2.9 Detection of Nrf2 Nuclear Translocation by Immunofluorescence

Immunofluorescence was used to determine the effect of EGCG on Nrf2 nuclear translocation. After treatments, the cells were washed three times with PBS and fixed with 4% paraformaldehyde at room temperature for 15 min, followed by permeabilization with 0.5% Triton X‐100 in PBS for 2 min on ice. After blocking, cells were incubated with primary antibody against Nrf2 (1:200 dilution) overnight at 4°C. Cells were incubated with anti‐rabbit secondary antibody labeled with Fluor‐488 for 1 h at room temperature. After washing, DAPI staining solution was added and incubated at room temperature in the dark for 10 min and the cells were observed under a fluorescence inverted microscope to assess the nuclear translocation of Nrf2.

### 2.10 Western Blot Analysis

Skin tissues or NHEK cells were lysed using RIPA, followed by protein separation via SDS‐PAGE and then transferred to PVDF membranes. Immunoblotting was performed using specific primary antibodies: anti‐Keap1 (Affinity, catalog number: AF5266, 1:2000), anti‐Nrf2 (Proteintech Group, catalog number: 16396‐1‐AP, 1:2000), anti‐HO‐1 (Proteintech Group, catalog number: 10701‐1‐AP, 1:1000), anti‐Histone H3 (Rui‐Ying Biological, catalog number: RLM3038, 1:30000), anti‐β‐actin (Proteintech Group, catalog number: 66009‐1‐Ig, 1:20000). Detection was achieved using a chemiluminescence‐based detection system for color development.

### 2.11 Molecular Docking

Molecular docking was further performed using AutoDock Vina to simulate the binding of keap1 and Nrf2 or EGCG to keap1‐Nrf2. Prior to conducting molecular docking, the 3D structure of EGCG was downloaded from PubChem, and the crystal structures of Keap1, Nrf2, and the Keap1‐Nrf2 binary complex (PDB ID: 3ZGC) were obtained from the RCSB PDB. After adding hydrogens and assigning partial charges, EGCG was prepared as the ligand and saved in pdbqt format, while the proteins were processed identically and saved as receptor pdbqt files. Fifty independent docking runs were performed and the most frequently observed pose with the lowest binding free energy was selected as the final model and visualized in PyMOL.

### 2.12 Statistical Analysis

The experimental data were analyzed using SPSS 23.0 statistical software. The results were expressed as mean ± standard deviation. One‐way analysis of variance (ANOVA) was used for multiple group comparisons, and the LSD post hoc test was applied as the samples met the assumptions of normality and homoscedasticity (Shapiro–Wilk test *p* > 0.05, Levene's test *p* > 0.05). A *p*‐value of less than 0.05 was considered statistically significant. Lesion scoring, histological counting behavior assessment and WB densitometry were independently performed by two blinded observers.

## 3 Results

### 3.1 EGCG Alleviated AD‐Like Symptoms Induced by DNFB

**FIGURE 1:**
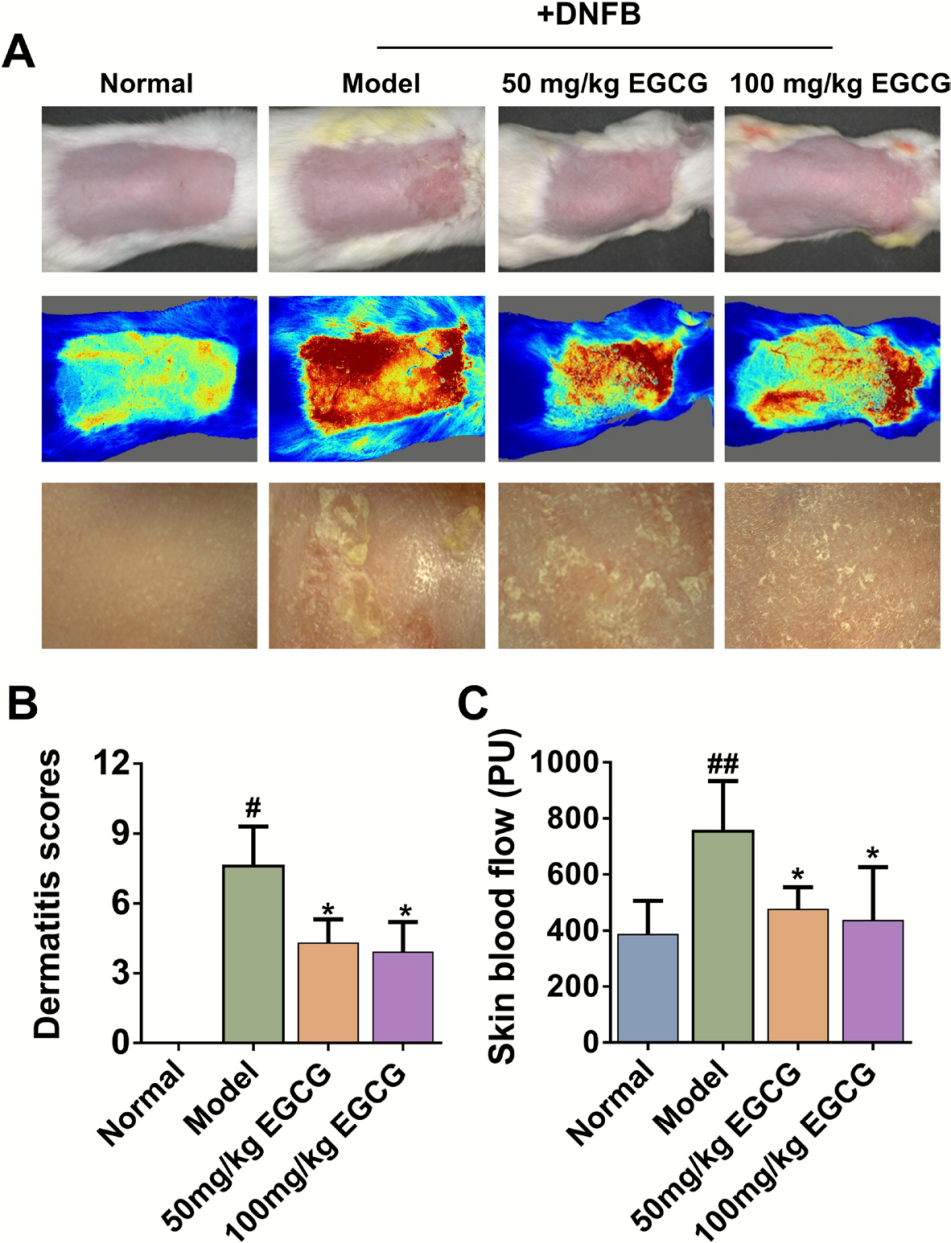
EGCG efficiently alleviated AD‐like symptoms induced by DNFB. (A) Representative photograph of dorsal skin and skin blood perfusion among different groups on Day 13. (B) Clinical skin severity scores for each group on Day 13. (C) The analysis of skin microvascular blood flow (PU) in each group. All data are expressed as mean ± SD (*n* = 8). ^#^Indicates *p* < 0.05 and ^##^indicates *p* < 0.01 as compared to the Normal group, *indicates *p* < 0.05 as compared to the Model group.

Compared to the normal group, DNFB‐induced AD mice exhibited obvious dry, crusted skin lesions with a marked increase in lesion scores (Figure 1A). Compared to the AD model group, 100 mg/kg EGCG significantly reduced the dermatitis scores from (7.60 ± 1.70) to (3.90 ± 1.30) (Figure 1B).

Laser Doppler flowmetry is now a well‐established, excellent noninvasive technique for measuring microvascular blood perfusion (Rajan et al. 2009; Sullender et al. 2002). In an inflamed state, the vasodilation and increased blood flow trigger the sharp increase of skin blood perfusion, thus leading to the skin erythema. Compared to the normal group, DNFB‐induced AD mice significantly increased the skin blood perfusion from (385.46 ± 120.43) to (754.14 ± 179.49) PU. Oral administration of 50 mg/kg EGCG decreased the skin blood perfusion to (474.94 ± 79.73) PU, while 100 mg/kg EGCG decreased that to (435.52 ± 190.54) PU (Figure 1C).

### 3.2 EGCG Ameliorated DNFB‐Induced Skin Swelling and Disrupted Skin Barrier

**FIGURE 2:**
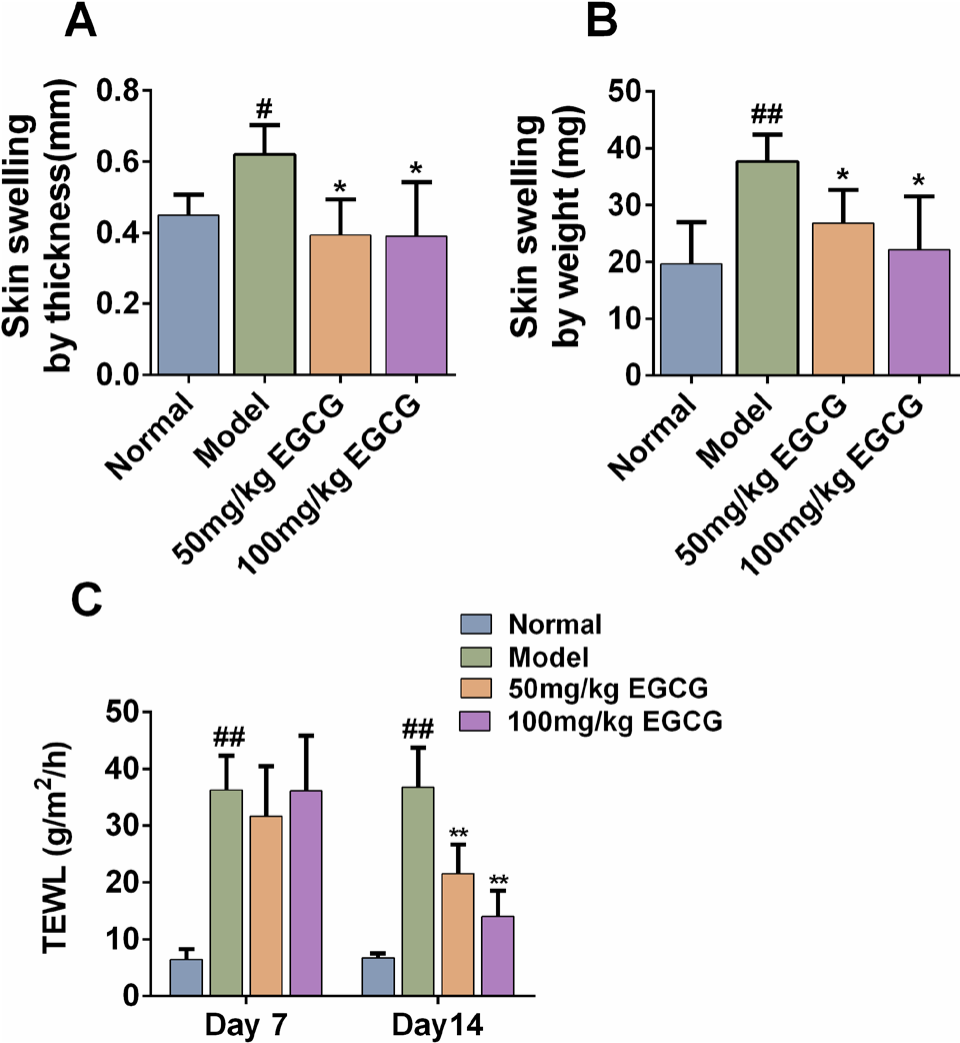
EGCG ameliorated DNFB‐induced skin swelling and disrupted skin barrier. (A) Skin swelling by thickness (mm) was measured on Day 13. (B) Skin swelling by weight (mg) was calculated on Day 13. (C) TEWL (g/m^2^/h) was measured on day 13. ^#^Indicates *p* < 0.05 and ^##^indicates *p* < 0.01 as compared to the Normal group, * indicates *p* < 0.05 and ** indicates *p* < 0.01 as compared to the Model group.

Increased skin thickness and impaired skin barrier function are typical skin manifestations of AD (Schmuth et al. 2024). We further assessed the therapeutic effect of EGCG on AD by measuring the skin swelling by thickness/weight and TEWL. As illustrated in Figure 2A,B, compared with the normal group, AD mice showed thickened dorsal skin, manifested as an increase in the thickness and weight of skin discs with the same area. In terms of skin swelling in thickness, as compared with the AD model group, the skin swelling decreased from (0.62 ± 0.08) to (0.39 ± 0.10) or (0.39 ± 0.15) mm by orally treating with 50 or 100 mg/kg EGCG (*p* < 0.05). A similar trend was also seen in skin swelling by weight.

In addition, the TEWL value of dorsal skin in the AD model group mice markedly elevated at Days 7 and 14. With the improvement of skin lesions, the TEWL in the 50 or 100 mg/kg EGCG group gradually decreased (Figure 2C). On Day 14, as compared with the AD model group, oral administration with 50 mg/kg EGCG inhibited the TEWL value from (36.78 ± 6.90) to (21.56 ± 5.09) g/m^2^/h, while 100 mg/kg EGCG decreased the value to (14.00 ± 4.51) g/m^2^/h.

### 3.3 EGCG Significantly Ameliorated the Pathological and Immunological Changes of AD Mice

**FIGURE 3:**
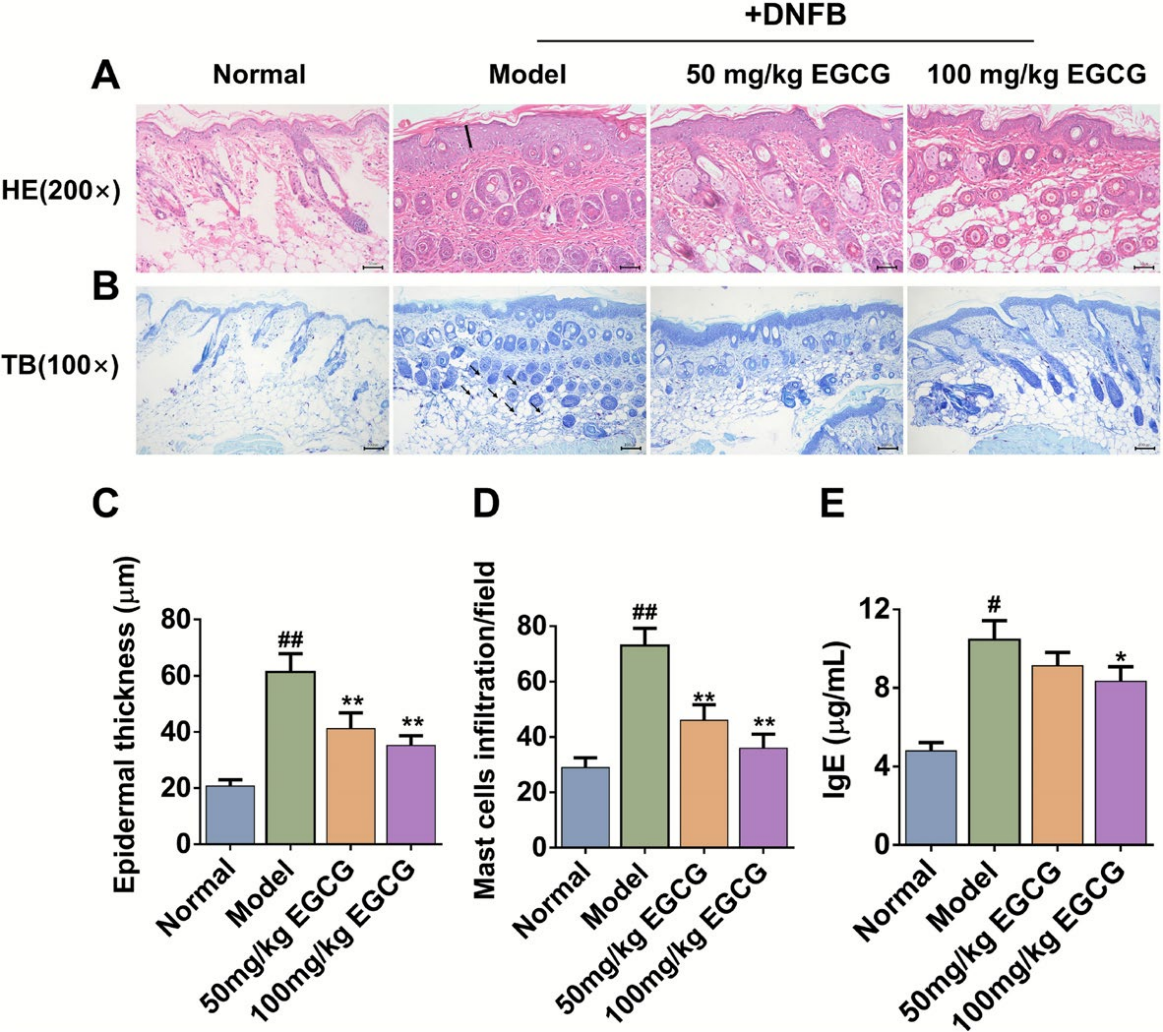
Effects of EGCG on DNFB‐induced epidermal hyperplasia and mast cell infiltration. (A) H&E staining (200× magnification, scale bars = 50 μm), the straight line indicates thickness of the epidermis. (B) Toluidine blue staining (100 × magnification, scale bars = 100 μm), the black arrow indicates mast cells in the skin stained with TB (C) Epidermal thickness (μm). (D) Mast cell count. (E) Quantification of IgE in serum. All data are expressed as mean ± SD (*n* = 8). ^#^Indicates *p* < 0.05 and ^##^indicates *p* < 0.01 as compared to Normal group, *indicates *p* < 0.05 and **indicates *p* < 0.01 as compared to Model group.

Patients with AD usually have elevated levels of IgE, and the level of IgE is correlated with the severity of AD to a certain extent (Hu et al. 2020). HE staining of skin sections can clearly show the structure and pathological changes of skin tissue, such as epidermal thickness and dermal inflammatory cell infiltration. TB staining reflects the number and distribution of mast cells in the skin lesion tissue. By combining IgE, HE and TB staining, we further evaluated the immunological and histopathological features among different groups. HE/TB staining revealed that AD mice were characterized by significant epidermal hyperplasia and infiltration of inflammatory cells and mast cells. Consistently, quantitative analysis of epidermal thickness showed that compared with the normal group, the epidermal thickness increased from (20.78 ± 2.26) to (61.40 ± 6.50) μm. TB staining showed that the number of mast cells increased from (29.00 ± 3.50) to (73.00 ± 6.20) cells/field. EGCG greatly reversed the above indicators in a dose‐dependent manner (Figure 3A,B, *p* < 0.01 or *p* < 0.05).

In addition, as compared with the normal group, the total IgE level in AD mice increased by 2.32 times, while EGCG at a dose of 100 mg/kg significantly reduced the total serum IgE level from (10.46 ± 0.97) to (8.33 ± 0.74) μg/mL (Figure 3C, *p* < 0.05).

### 3.4 EGCG Alleviated the Comorbid Anxiety‐ and Depressive‐Like Behaviors in AD Mice

**FIGURE 4:**
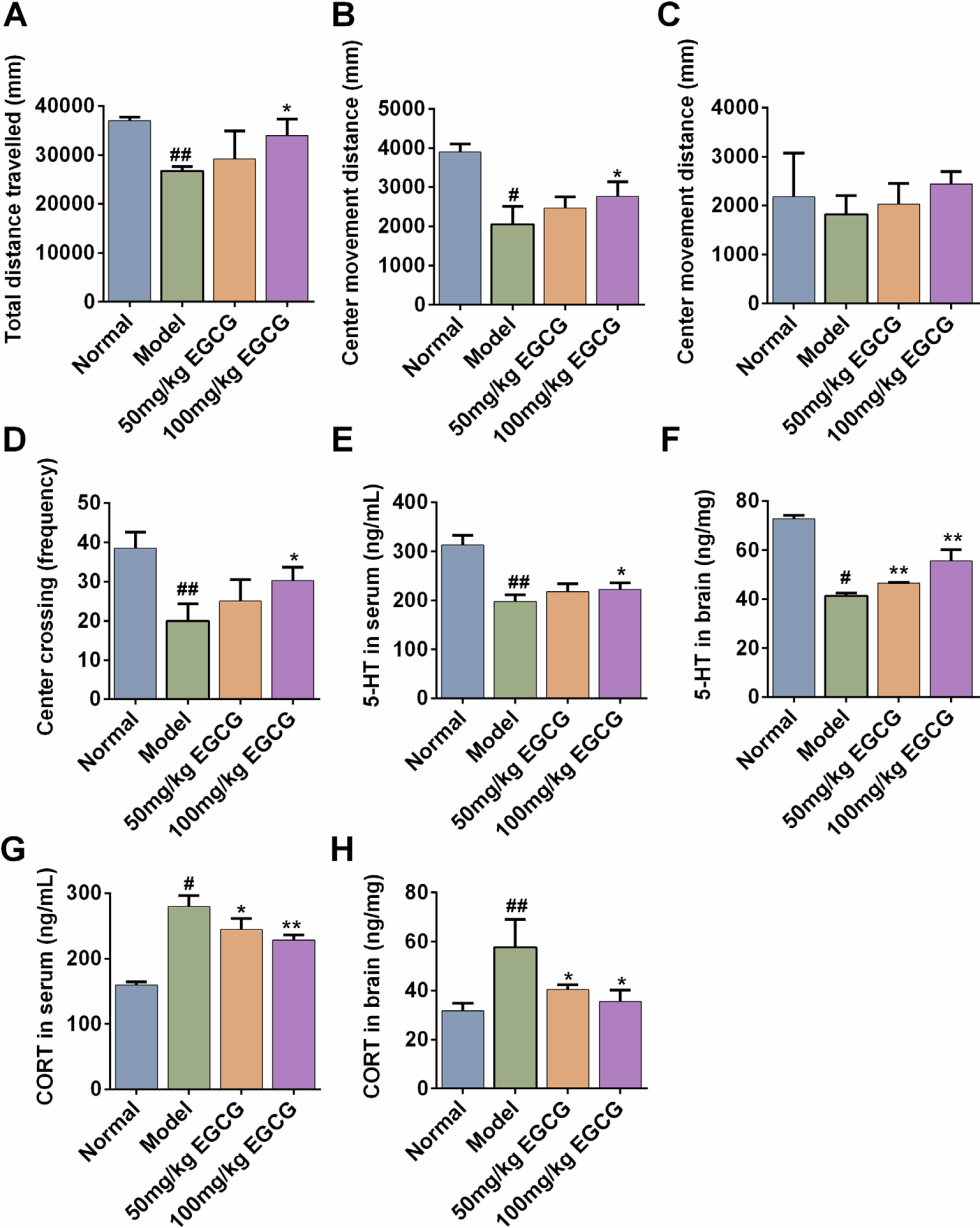
EGCG alleviated the psychiatric comorbidities in AD mice. (A) Total distance traveled (mm) in OFT. (B) The distance traveled in the central area (mm) in OFT. (C) The central distance traveled (mm) in EPM. (D) The frequency of entering into the central area in EPM. (E) Serum level of 5‐HT. (F) Brain level of 5‐HT. (G) Serum level of CORT. (H) Brain level of CORT. All data are expressed as mean ± SD (*n* = 8). ^#^Indicates *p* < 0.05 and ^##^indicates *p* < 0.01 as compared to the Normal group, *indicates *p* < 0.05 and **indicates *p* < 0.01 as compared to the Model group.

The OFT and the EPM were employed to quantify general locomotor activity, and anxiety‐ and depressive‐like behaviors. In the OFT, AD mice in the model group displayed marked hypolocomotion and avoidance of the aversive center, evidenced by reduced total distance and central zone distance (*p* < 0.01 or *p* < 0.05), reflecting the diminished exploratory drive. Administration of 100 mg kg^−1^ EGCG restored both indices to control levels, indicating an anxiolytic action (Figure 4A,B). In the EPM, AD mice made fewer central‐platform entries, and EGCG increased the frequency traveled on the central platform, reflecting enhanced risk‐assessment/exploratory motivation (*p* < 0.01 or *p* < 0.05). The distance traveled in the central area of AD mice exhibited a similar trend, although there was no statistical difference (Figure 4C,D).

### 3.5 EGCG Modulated the Neurotransmitter Levels in AD Mice

5‐hydroxytryptamine (5‐HT), a monoamine neurotransmitter, is pivotal in the regulation of the central nervous system. Impairments in the serotonergic system are linked to behaviors indicative of depression (Pourhamzeh et al. 2022). As depicted in Figure 4E, the 5‐HT concentration in the serum of AD mice prominently declined compared with that in the Normal group (313.26 ± 11.61 ng/mL vs. 197.59 ± 13.82 ng/mL, *p* < 0.01). Oral administration with 100 mg/kg EGCG remarkably restored the decline (222.51 ± 13.11 ng/mL, *p* < 0.05). Similar changing trends were also found in brain levels of 5‐HT (Figure 4F).

CORT is the hormonal end product of the HPA axis and the elevated CORT levels are characteristic of the pathophysiology of chronic stress and depressive disorder (Ma et al. 2023; Spencer and Deak 2017). In our study, the level of CORT in the brain/serum was remarkably increased in the AD Model group compared with those in the Normal group (*p* < 0.01, Figure 4G,H). In comparison to the AD Model group, the serum level of CORT was dose‐dependently decreased in the 50 mg/kg (279.70 ± 16.84 ng/mL vs. 244.52 ± 16.52 ng/mL, *p* < 0.01) or 100 mg/kg (279.70 ± 16.84 ng/mL vs. 228.10 ± 8.35 ng/mL, *p* < 0.01) EGCG group (Figure 4G). In addition, 50 or 100 mg/kg EGCG treatment inhibited the increased brain CORT level in AD mice (Figure 4H, *p* < 0.05).

### 3.6 EGCG Attenuated Oxidative Damage in AD Mice

**FIGURE 5:**
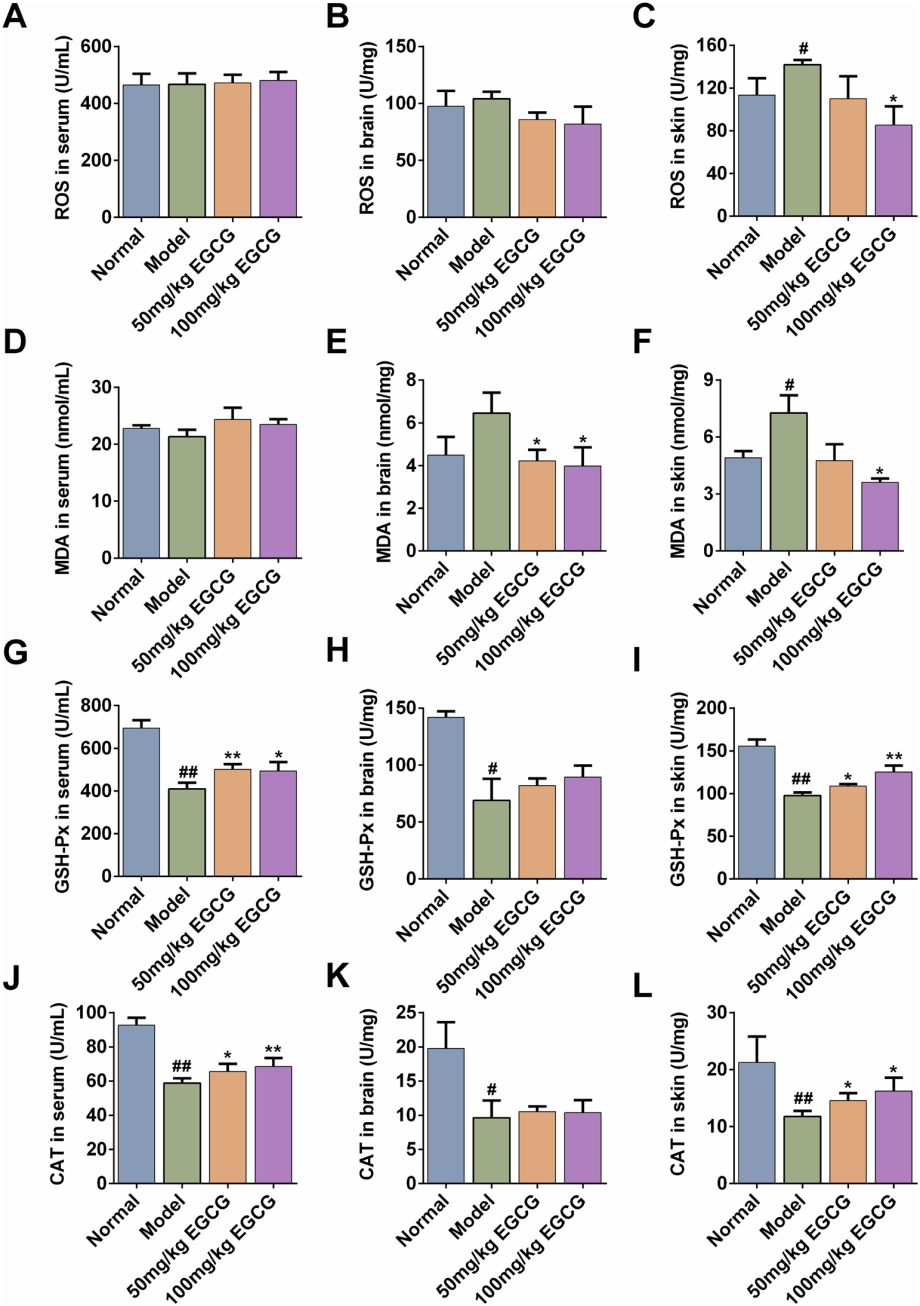
Effects of EGCG on the oxidative stress levels in DNFB‐induced mice. Serum/brain/skin of ROS (A–C), or MDA (D–F), or GSH‐Px (G–I), and CAT (J–L) were determined. All data are expressed as mean ± SD (*n* = 5). ^#^Indicates *p* < 0.05 and ^##^indicates *p* < 0.01 as compared to the Normal group, * Indicates *p* < 0.05 and **indicates *p* < 0.01 as compared to the Model group.

During oxidative stress, ROS, MDA, GSH‐Px, CAT are different substances or enzymes, which are collectively maintaining the intracellular redox equilibrium and safeguarding cells from oxidative damage via distinct mechanisms (Demirci‐Çekiç et al. 2022; Marrocco et al. 2017). To assess the effect of EGCG on the levels of oxidative stress in AD, we further examined the levels of ROS, MDA, GSH‐Px, CAT in the serum/brain/skin. In serum and brain tissue, we did not observe evident changes in the levels of ROS (Figure 5A,B, *p >* 0.05). Yet, compared to the control group, the skin ROS level was significantly increased from (113.43 ± 15.64) to (141.83 ± 4.47) U/mg. The main reason was probably due to the transient nature of ROS. Repeated DNFB to the dorsal skin confined the ROS burst to the local lesions, whereas any ROS that entered the circulation or brain had already been scavenged before collection. Oral administration of 100 mg/kg EGCG markedly attenuated the ROS levels in the skin (Figure 5C, *p* < 0.05). In addition, the increase of MDA level in the brain/skin was significantly decreased by 100 mg/kg EGCG, whereas no significant difference in serum MDA level was observed among all groups (Figure 5D,F).

On the contrary, the serum/skin levels of GSH‐Px and CAT were both significantly decreased in the DNFB‐induced AD group (Figure 5G,I,J,L, *p* < 0.01 or *p* < 0.05). Treatment with 50 or 100 mg/kg could significantly restore the GSH‐Px and CAT levels to near normal values (*p* < 0.05 or *p* < 0.01). Unfortunately, no significant difference was observed in brain levels of GSH‐Px and CAT between Model and EGCG‐treated groups (Figure 5H,K).

### 3.7 EGCG Activated the Keap1/Nrf2/HO‐1 Signaling Pathway in Dorsal Skin

**FIGURE 6:**
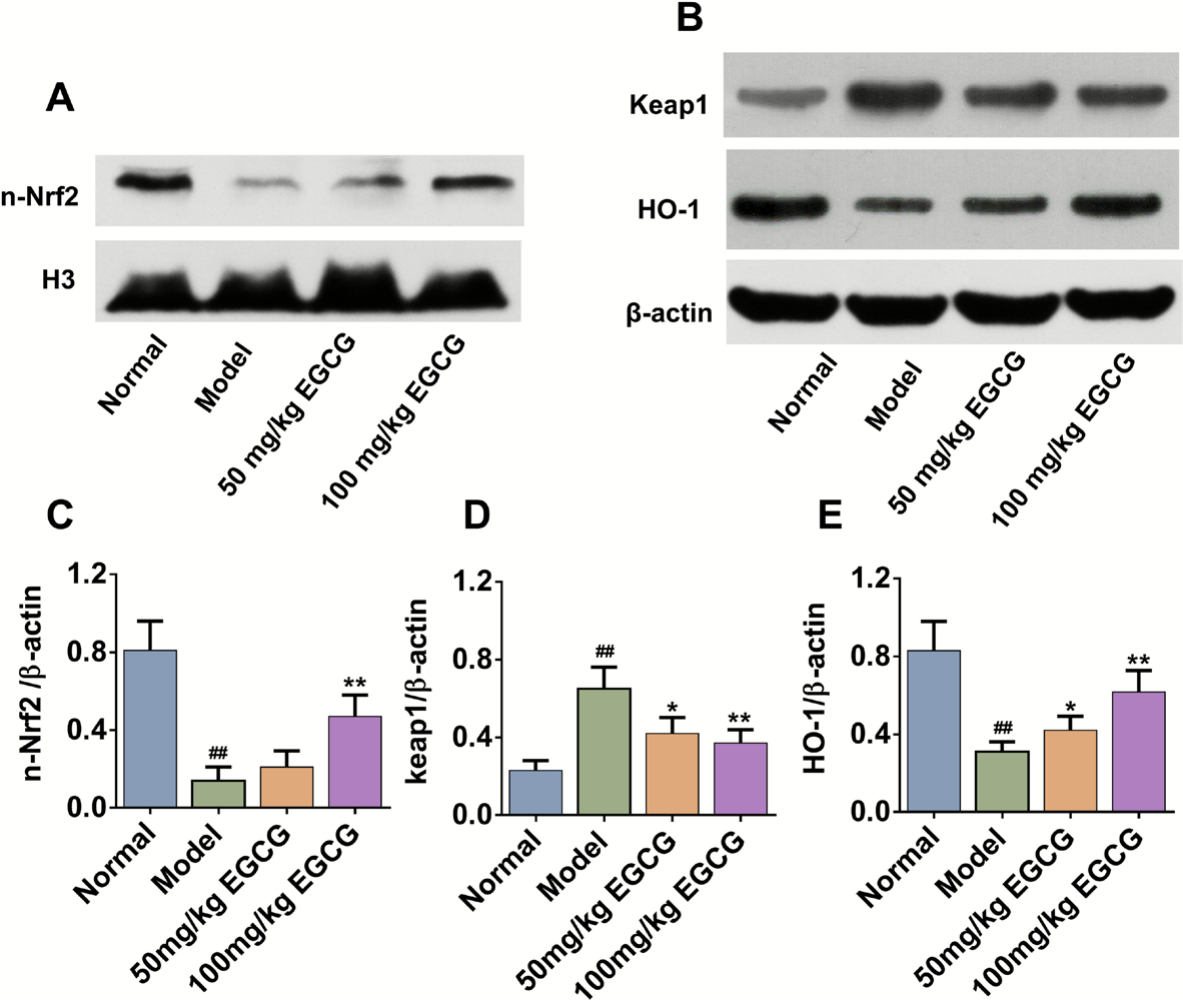
EGCG activated the Keap1/Nrf2/HO‐1 signaling pathway in dorsal skin. Western blot for (A) n‐Nrf2, (B) Keap1 and HO‐1. Relative protein levels of (C) n‐Nrf2, (D) Keap1 and (E) HO‐1 were normalized to H3 or β‐Actin, respectively. All data are expressed as mean ± SD (*n* = 3). ^#^Indicates *p* < 0.05 and ^##^indicates *p* < 0.01 as compared to the Normal group, *indicates *p* < 0.05 and **indicates *p* < 0.01 as compared to the Model group.

The activation of the Keap1/Nrf2/HO‐1 pathway is generally involved in regulating intracellular oxidative stress and scavenging ROS. Under normal physiological conditions, Nrf2 resides in the cytoplasm and associates with Keap1, helping to preserve redox balance. When oxidative stress occurs, Nrf2 moves to the nucleus, where it promotes the transcription and expression of antioxidant genes such as HO‐1 and SOD (Suzuki et al. 2023). The protein expressions of nuclear Nrf2, and Keap1/HO‐1 in the cytoplasm were further examined, respectively. As is shown in Figure 6, the results showed that compared with the Normal group, the expression of nuclear Nrf2 protein and total HO‐1 protein in the skin tissues of the Model group mice was significantly decreased, while the expression of total Keap1 protein was increased. EGCG increased the expression of nuclear Nrf2 protein and total HO‐1 protein, and decreased the expression of total Keap1 protein (*p* < 0.01 or *p* < 0.05).

### 3.8 EGCG Inhibited the Production of ROS in TNF‐α/IFN‐γ‐Induced NHEK Cells

NHEK cells stimulated with TNF‐α and IFN‐γ are extensively utilized to study AD‐like responses (Luo et al. 2022). To delve deeper into the protective mechanisms of EGCG in AD, an AD cell model induced by TNF‐α/IFN‐γ was established. Firstly, NHEK cells were exposed to various concentrations of EGCG ranging from 1 to 200 μM for 24 h using a CCK‐8 assay to identify the optimal concentration of EGCG. The findings revealed that EGCG concentrations below 25 μM were noncytotoxic to NHEK cells. Moreover, EGCG at concentrations below 25 μM did not adversely affect the growth and development of TNF‐α/IFN‐γ‐stimulated NHEK cells (Figure S2). The TNF‐α/IFN‐γ‐stimulated NHEK cells were then randomly assigned into five groups: control, AD, and AD + EGCG (5, 10, 25 μM).

**FIGURE 7:**
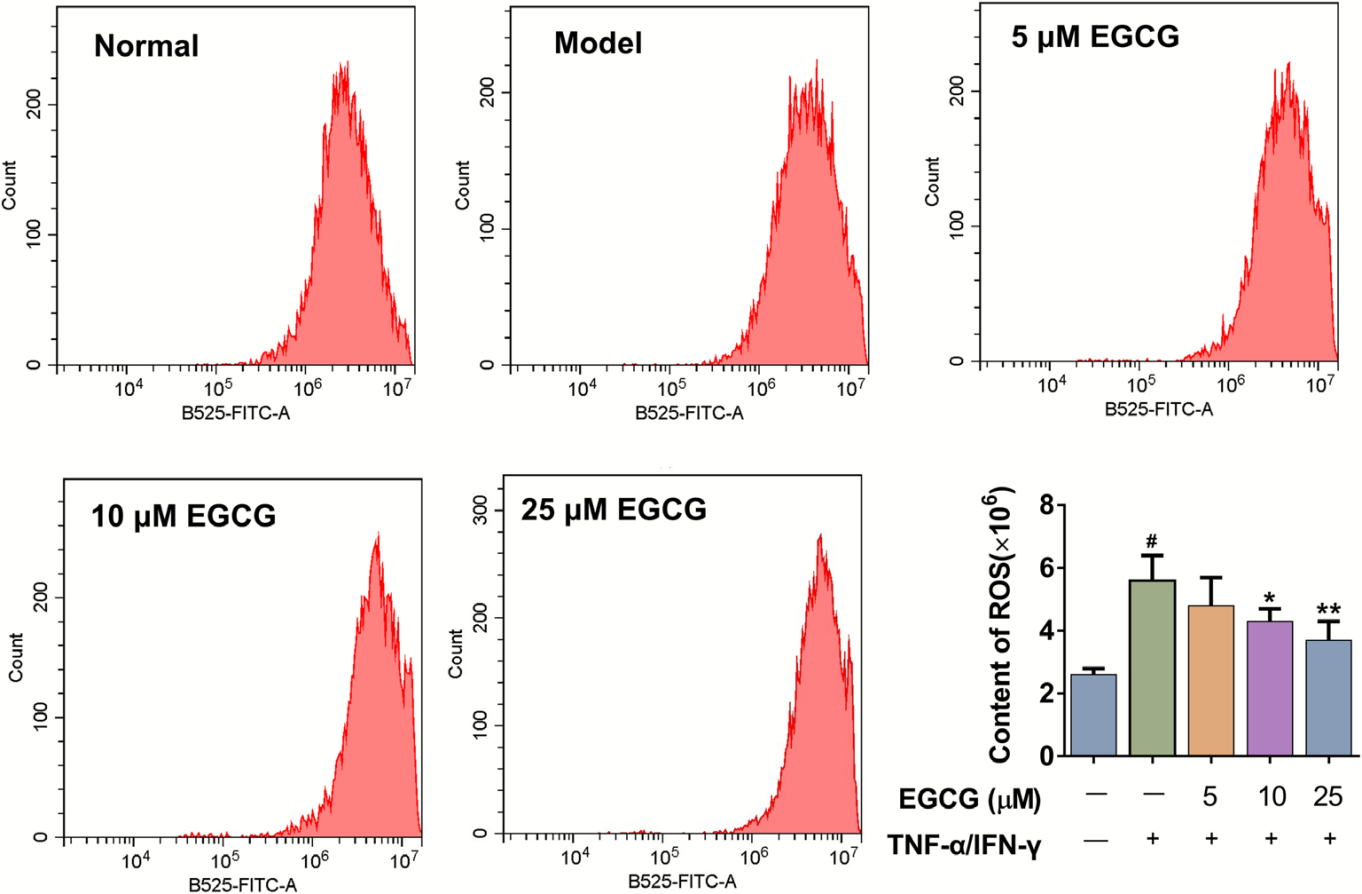
Effects of EGCG on the production of ROS in TNF‐α/IFN‐γ‐induced NHEK cells using flow cytometry. All data are expressed as mean ± SD (*n* = 3). ^#^Indicates *p* < 0.05 as compared to the Normal group, *indicates *p* < 0.05 and **indicates *p* < 0.01 as compared to the Model group.

To examine the effect of EGCG on oxidative damage in the AD cell model, the production of ROS was detected using flow cytometry with the DCFH‐DA probe. As shown in Figure 7, the ROS level in the TNF‐α/IFN‐γ‐stimulated NHEK cells group was significantly elevated compared to that in the control group (*p* < 0.05). Compared with the AD group, EGCG dose‐dependently inhibited the release of ROS (*p* < 0.01 or < 0.05).

### 3.9 EGCG Promoted the Nuclear Translocation of Nrf2 in TNF‐α/IFN‐γ‐Induced NHEK Cells

**FIGURE 8:**
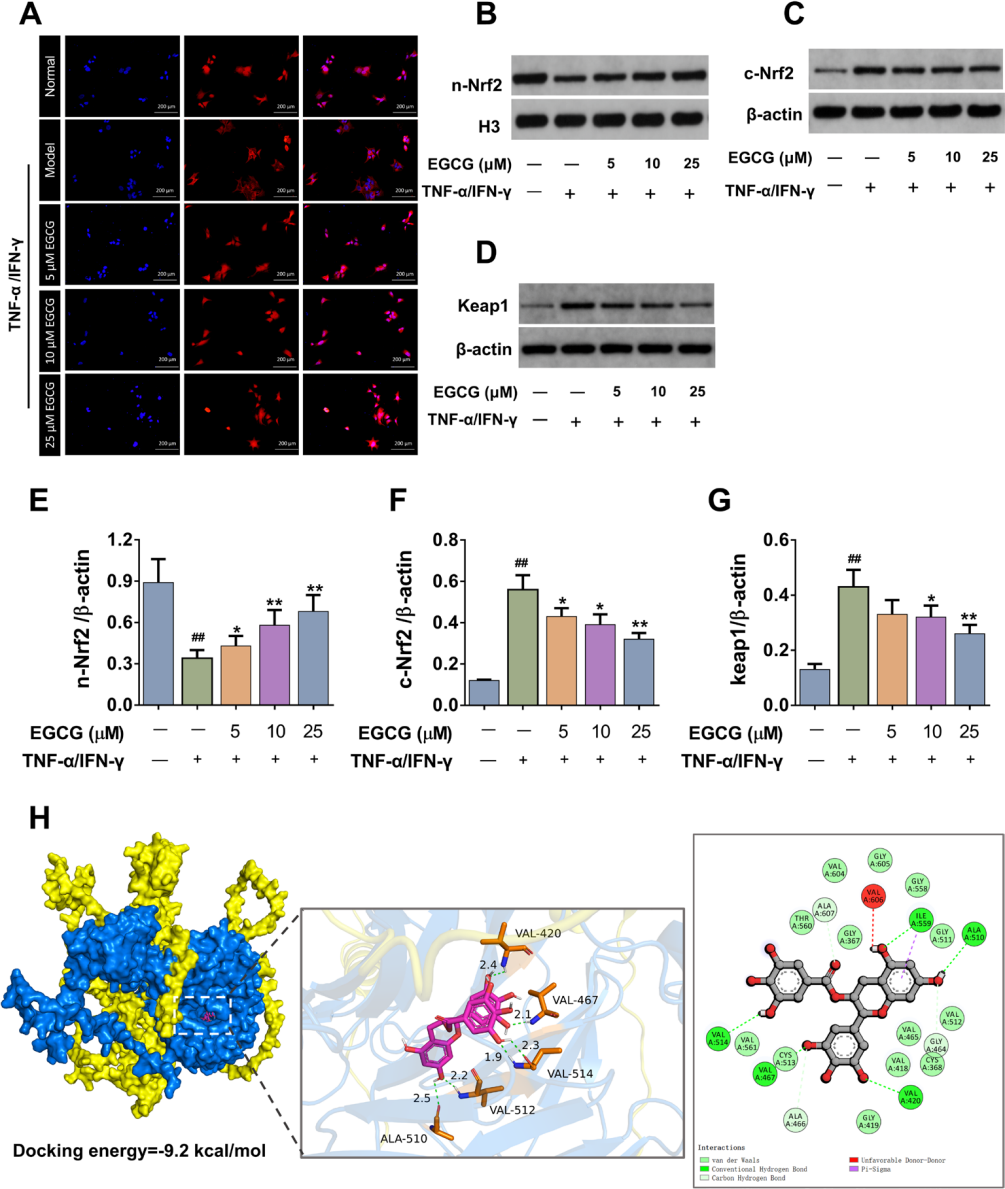
EGCG binds Keap1 to promote the nuclear translocation of Nrf2 in vitro. (A) Immunofluorescence staining. Western blot for (B) n‐Nrf2, (C) c‐Nrf2, and (D) Keap1. Relative protein levels of (E) n‐Nrf2, (F) c‐Nrf2 and (G) Keap1 were normalized to H3 or β‐Actin. (H) The optimal molecular docking conformations of EGCG with the Keap1/Nrf2 binary complex. All data are expressed as mean ± SD (*n* = 3). ^##^indicates *p* < 0.01 as compared to the Normal group, *indicates *p* < 0.05 and **indicates *p* < 0.01 as compared to the Model group.

Under oxidative stress, Nrf2 is transferred to the nucleus and induces the transcription and expression of HO‐1 and SOD (Karunatilleke et al. 2021). Immunofluorescence and western blotting were employed to examine the nuclear translocation of Nrf2 in NHEK cells. Compared with normal NHEK cells, the level of Nrf2 protein in the nucleus slightly decreased after TNF‐α/IFN‐γ stimulation. EGCG intervention could significantly enhance the nuclear translocation of Nrf2 and increase the protein expression of HO‐1 and nuclear Nrf2 (Figure 8A–G). Taken together, EGCG could efficiently activate the Keap1/Nrf2/HO‐1 signaling pathway, thereby promoting the expression of antioxidant enzymes (e.g., HO‐1 and SOD) and inhibiting ROS accumulation to protect cells from oxidative stress‐induced damage.

### 3.10 EGCG Probably Binds to Keap1 Inhibit the Interaction of the Keap1/Nrf2 Binary Complex

To gain initial insight into how EGCG drives Nrf2 nuclear translocation, we performed molecular docking to map the amino acid residues and intermolecular forces that EGCG engages on the Keap1–Nrf2 binary complex. Molecular docking analysis revealed that Keap1 and Nrf2 form a stable binary complex (binding energy = −20.8 kcal/mol, Figure S3). When EGCG was docked into the binary complex, it bound exclusively to Keap1, establishing five hydrogen bonds with amino acid residues of Val‐420, Val‐512, Val‐514, Val‐467 and Ala‐510 (Figure 8H). The free energy of the optimal docking conformation between EGCG and Keap1–Nrf2 showed a markedly weakened (binding energy = −9.2 kcal mol^−1^), implying that EGCG can allosterically disrupt the complex and thereby facilitate Nrf2 nuclear entry.

## 4 Discussion

AD is a highly prevalent, chronic relapsing inflammatory dermatosis that often coexists with mental disorders, which amplify disease burden and profoundly impair health‐related quality of life. Accumulating evidence has demonstrated that oxidative stress is a key driver of AD pathophysiology (Liu et al. 2023). Excessive ROS generated by oxidative imbalance up‐regulate NLRP3 inflammasome activation, amplifying the release of IL‐1β and other pro‐inflammatory cytokines, exacerbating pruritus, and eroding stratum‐corneum integrity. The consequent barrier disruption further facilitates deeper penetration of allergens and microbes, perpetuating cutaneous inflammation (Raimondo et al. 2023; Wang et al. 2020). Simultaneously, oxidative stress triggers canonical inflammatory cascades such as NF‐κB, which in turn disrupt neurotransmitter homeostasis, foster neuroinflammation, and impair synaptic plasticity, ultimately driving depressive‐ and anxiety‐like behaviors (Correia et al. 2023). EGCG, the primary polyphenolic compound in green tea, possesses well‐documented antioxidant, anti‐inflammatory, and anti‐oxidative properties, indicating its therapeutic potential in AD. Taking oxidative stress as the breakthrough, we therefore investigated whether EGCG could attenuate AD‐like skin manifestations and AD‐associated psychiatric comorbidities in vitro and in vivo.

In the study, repetitive topical exposure of DNFB on the mouse skin consistently reproducibly elicited AD‐like phenotypes characterized by scaling, erythema, and excoriation, closely recapitulating human pathology. Moreover, DNFB‐induced AD mice exhibited elevated dermatitis scores, skin thickening, skin blood perfusion, TEWL and serum levels of IgE. Dermal edema and elevated cutaneous blood flux are objective biomarkers of skin inflammation. A localized rise in perfusion indicates arteriolar and capillary dilatation within the superficial vascular plexus, which is clinically expressed as erythema (Clough 1999; Clough and Church 2002). TEWL serves as a quantitative biomarker of epidermal barrier competence and elevated values indicate progressive barrier dysfunction (Cho et al. 2025). Orally administered doses of 50 or 100 mg/kg EGCG significantly ameliorated cutaneous lesions, prevented epidermal hyperplasia and recovered disrupted skin barrier. The infiltration of inflammatory cells and mast cells in cutaneous tissues was also effectively inhibited by EGCG.

Individuals afflicted with AD are prone to develop comorbid anxiety and/or depression, which reciprocally amplify pruritus and lesion severity, establishing a self‐perpetuating neuro‐psycho‐dermatological loop (LeBovidge and Schneider 2025). To comprehensively assess changes in psychological behaviors, OFT and EPM were performed in this study. AD mice displayed evident psychological stress and heightened anxiety, as demonstrated by the diminished exploratory drive, spontaneous locomotion, open‐arm exploration and central‐platform occupancy. Concordantly, AD mice in the Model group exhibited a pronounced depletion of 5‐HT and elevated CORT in serum/brain, implicating disrupted neurotransmitter metabolism and hyperactivation of the HPA axis in the genesis of AD‐associated depressive states. However, EGCG greatly normalized the anxiety‐ and depressive‐like behavioral endophenotypes and reinstated neurotransmitter homeostasis, providing robust evidence that mitigation of AD‐equivalent cutaneous pathology is coupled with a concomitant reduction in its attendant psychiatric comorbidities.

ROS and MDA (a terminal metabolite of lipid peroxidation), together with the antioxidant enzymes, such as CAT and GSH‐Px, are quantifiable surrogates of redox equilibrium. An increment in ROS/MDA generation or a parallel decline in CAT/GSH‐Px activity unequivocally signifies intensified oxidative stress (Hassan et al. 2024). Skin GSH‐Px and CAT decompose hydrogen peroxide into water and oxygen, thereby reducing oxidative stress and protecting skin cells (He et al. 2024). In the DNFB‐induced AD model group, elevated oxidative‐stress levels were observed in the skin/serum/brain of AD mice. Compared to the AD model group, EGCG reduced MDA content and ROS accumulation in the skin/serum/brain, while enhancing GSH‐Px and CAT activity (*p* < 0.05 or *p* < 0.01). Literature reported (Wang et al. 2020; Rawani et al. 2024) that neurotransmitter dysregulation and exaggerated oxidative stress represent fundamental pathobiological alterations underpinning depression and anxiety, while simultaneously functioning as key amplifiers of cutaneous inflammatory responses. Collectively, we propose that EGCG reinstates a dysregulated neuro‐cutaneous‐immune axis in AD mice by systemically suppressing ROS‐mediated oxidative stress, concomitantly mitigating epidermal lesions and comorbid anxiety‐and depressive‐like symptoms.

To further explore the potential anti‐oxidative stress mechanism of EGCG in AD, we investigated its effects on the Keap1/Nrf2/HO‐1 signaling pathway. Under normal physiological conditions, Nrf2 primarily interacts with its inhibitor Keap1 and exists in the cytoplasm in an inactive state. Upon exposure to oxidative stressors such as ROS, Nrf2 is uncoupled from Keap1 and translocated into the nucleus to bind to antioxidant response elements (AREs), thereby regulating the expression of antioxidant enzyme genes, including reduced coenzyme II (NADPH), quinone oxidoreductase 1 (NQO1), HO‐1, etc. This process facilitates enhancing the resistance of cells and tissues to oxidative stress (Bellezza et al. 2018; Loboda et al. 2016). In our in vivo experiment, we found that EGCG inhibited KEAP1 expression and upregulated nuclear Nrf2 and HO‐1 protein expression in skin lesions. Consistently, in an in vitro AD model, EGCG remarkably inhibited the ROS accumulation and promoted nuclear translocation of Nrf2 in NHEK cells stimulated with TNF‐α/IFN‐γ. In addition, molecular docking between EGCG and the Keap1‐Nrf2 protein revealed that EGCG binds to Keap1, forming five hydrogen bonds with amino acid residues VAL‐420, VAL‐512, VAL‐514, VAL‐467, and ALA‐510. This suggested that EGCG might promote the dissociation of Nrf2 and Keap1 by competing for Nrf2‐binding sites on Keap1, enhancing Nrf2 nuclear translocation and reducing oxidative stress in AD. However, further experiments such as CETSA (cellular thermal shift assay), SPR (surface plasmon resonance), and mutagenesis analyses (e.g., substituting Val‐420, Val‐512, Val‐514, Val‐467, and Ala‐510 with nonconservative residues) were still required to fully understand how EGCG specifically disrupted the Keap1‐Nrf2 interaction and to delineate the importance of its binding site.

## 5 Conclusion

The present study authenticated for the first time that EGCG exhibited ameliorative properties against DNFB‐induced AD‐like skin lesions and comorbid anxiety and depression, and its reformative effects were likely partially mediated through the activation of the Keap1/Nrf2/HO‐1 signaling pathway, thereby relieving the excessive oxidative stress, altering the HPA axis dysfunction and upregulating the neurotransmitters. These data proved that EGCG holds significant promise as a candidate agent to treat AD and its comorbid anxiety and depression.

## Conflicts of Interest

The authors declare no conflicts of interest.

## Supporting information


**Figure S1:** Summary of the experimental process.

**Figure S2:** Effect of EGCG on viability of NHEKs cells. (A) Effect of different concentrations of EGCG on viability of NHEKs cells. (B) Effect of EGCG on viability of TNF‐α/IFN‐γ‐induced NHEK cells. All data are expressed as mean ± SD (*n* = 5). ^#^Indicates *p* < 0.05 and ^##^indicates *p* < 0.01 as compared to Normal group, *Indicates *p* < 0.05 and **indicates *p* < 0.01 as compared to Model group.

**Figure S3:** The optimal molecular docking conformations of Keap1 and Nrf2.

## Data Availability

The data that support the findings of this study are available on request from the corresponding author. The data are not publicly available due to privacy or ethical restrictions.
