# Supplementary figures and images for: Epigallocatechin‐3‐Gallate Mitigates Atopic Dermatitis‐Like Skin Lesions and Psychiatric Comorbidities by Reducing Oxidative Stress
Source: Food Sci Nutr. 2025 Nov 23;13(11):e71248. doi: 10.1002/fsn3.71248 (PMC12641101; doi:10.1002/fsn3.71248)

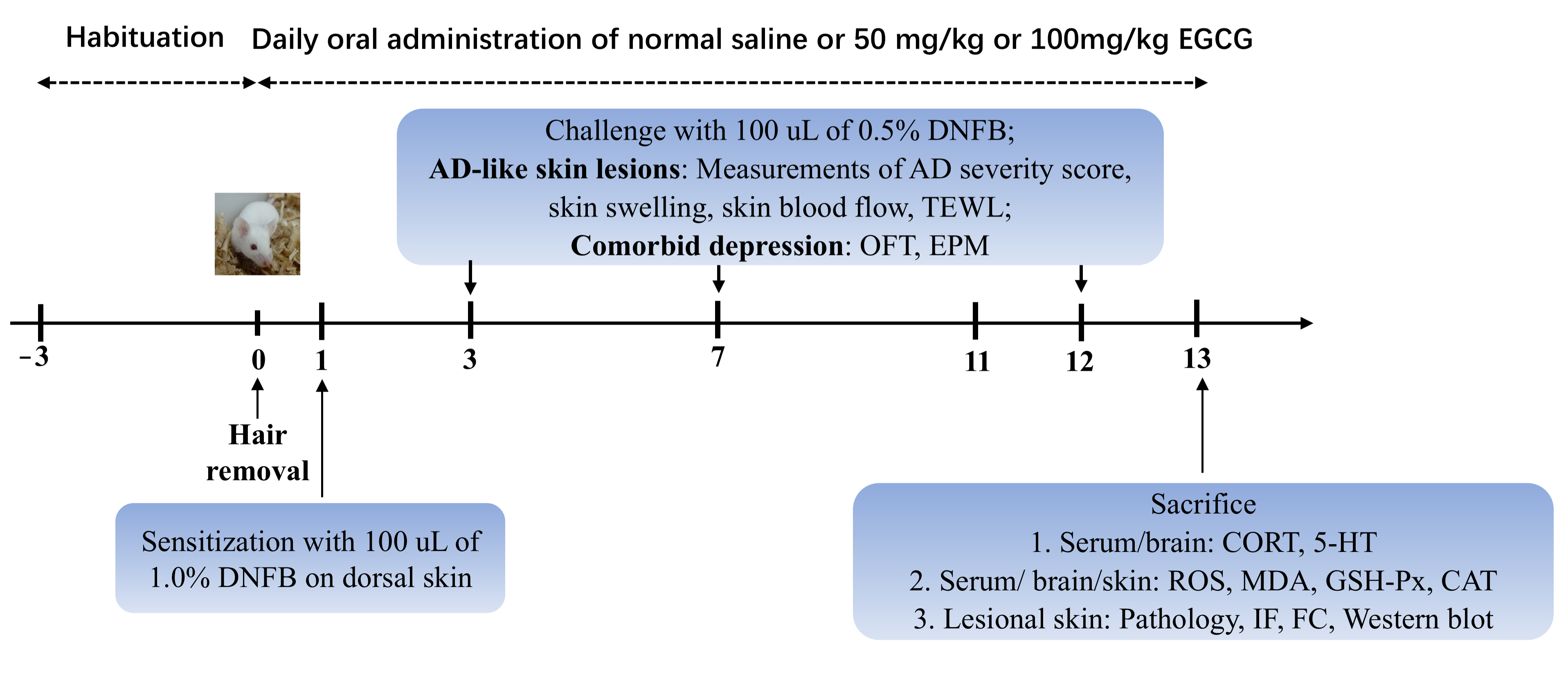

Supplement: Supplementary file 1 — Figure S1: Summary of the experimental process. [file FSN3-13-e71248-s001.tif]

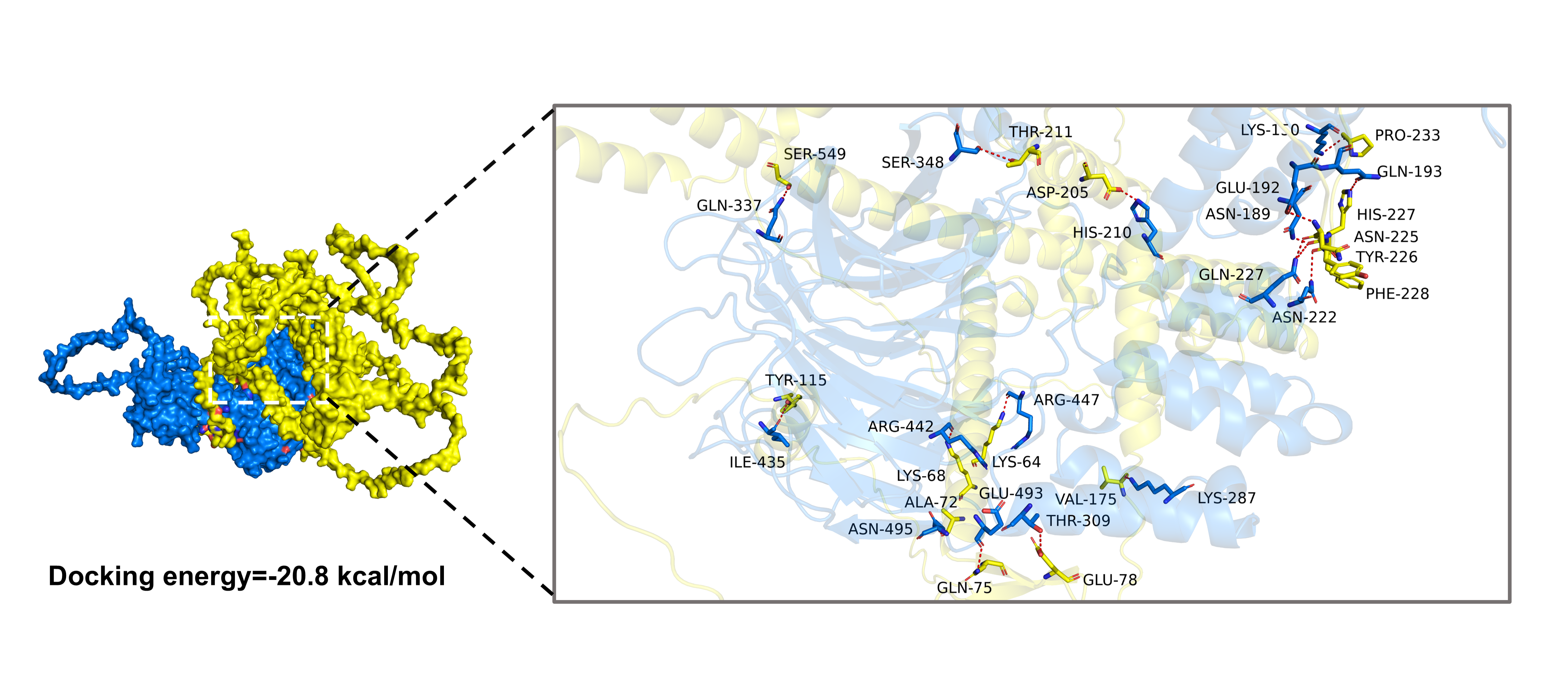

Supplement: Supplementary file 3 — Figure S3: The optimal molecular docking conformations of Keap1 and Nrf2. [file FSN3-13-e71248-s002.tif]
